# Targeting ferroptosis in osteoporosis: mechanisms and natural products therapies 

**DOI:** 10.3389/fphar.2026.1726949

**Published:** 2026-06-25

**Authors:** Zhanhong Cao, Chen Zhang, Xiaofan Yang, Yue Shen, Yuxin Bai

**Affiliations:** School of Traditional Chinese Medicine, Bozhou University, Bozhou, Anhui, China

**Keywords:** antioxidant regulation, ferroptosis, iron metabolism, lipid peroxidation, natural products, osteoporosis

## Abstract

Osteoporosis (OP) persists as the principal contributor to the global disease burden. OP is defined by reduced bone mass and impaired bone microarchitecture, thereby weakening bone strength and elevating fracture risk. Imbalanced bone remodeling is the major pathological mechanism of OP, in which osteoclast-mediated bone resorption exceeds osteoblast-mediated bone formation. Iron serves as a vital micronutrient for numerous biochemical activities and is essential for many cellular processes. Ferroptosis is an iron-dependent form of modulated cell death with unique traits, including disrupted iron balance, compromised antioxidant defenses, and aberrant lipid peroxidation. Ferroptosis participates in various physiological and pathological processes, and its role in bone-related diseases, particularly OP, is increasingly being explored. Hence, a systematic examination of the mechanisms modulating ferroptosis in OP is imperative to pinpoint potential therapeutic targets and innovate novel therapeutic and/or preventive strategies. The agents currently used to treat OP have numerous side effects, prompting increased research on natural compounds for OP treatment. This review systematically summarizes the key features and modulation mechanisms of ferroptosis based on the latest research advances, and explores its pathogenic implications and therapeutic opportunities in OP. Additionally, we also discussed investigations of natural products *in vitro* and *in vivo* to prevent OP by interfering with ferroptosis.

## 1 Introduction

Osteoporosis (OP) is a progressive bone disease defined by bone mass reduction and bone microarchitecture deterioration, which compromises bone strength and increases fracture risk (Zhang J. et al., 2023). OP is a global metabolic bone disease that impacts over 200 million people around the world (Ali and Kim, 2025). Of these individuals, roughly 50% of women and 20% of men will undergo fracture due to OP beyond the age of 50 (Lorentzon, 2019). Notably, the population of individuals aged 50 and above with OP is expected to exceed 400 million by 2050 owing to population aging and rising life expectancy (Liu J. et al., 2024). Most patients receive a definitive diagnosis only after suffering an osteoporotic fracture, resulting in varying degrees of diagnostic delay that has become a public health challenge due to its substantial impact on patient health and societal economic burden (Du et al., 2024). Bone remodeling is vital for maintaining bone homeostasis and involves a coordinated balance between osteoblast (OB)-driven bone formation and osteoclast (OC)-mediated bone resorption to ensure global mineral homeostasis and optimal bone mass (Gao et al., 2021; Chen J. et al., 2023). The balance is disrupted in OP due to either the suppression of OB function or the elevation of OC activity (Yokota et al., 2024). Growing evidence demonstrates that iron excess modulates the differentiation and activity of OB and OC in a way that disrupts the precise equilibrium between bone formation and resorption, thereby accelerating the process of bone loss (Ru et al., 2023). This implies that maintaining optimal iron levels is crucial for preserving bone homeostasis.

Iron is an indispensable trace element for various biochemical activities that are engaged in the growth of cells and tissues, and is pivotal in regulating energy metabolism, oxygen transport, and many cellular processes (Tao et al., 2024; Freitas et al., 2026). The iron homeostasis is precisely regulated under physiological conditions (Jin et al., 2021). Iron homeostasis disorders, both iron excess and iron deficiency, are closely related to OP (Che et al., 2020). Numerous clinical studies have shown that among iron overload disorders-including thalassemia, sickle cell disease, and hereditary hemochromatosis-a common feature is bone loss, which is marked by reduced bone mass, OP, and increased risk of bone fractures (Jeney, 2017). Furthermore, studies have confirmed that iron overload promotes bone resorption and inhibits bone formation by inhibiting OB differentiation, promoting OC generation, and increasing OC activity (Dong et al., 2024; Jiang J. et al., 2024). Numerous studies indicate that various diseases are associated with ferroptosis, including OP (Hu et al., 2024). Given that dysregulation of biological processes associated with ferroptosis (e.g., iron metabolism, lipid metabolism, and signaling pathways regulating ferroptosis) is tightly linked to the occurrence and progression of OP. Understanding the mechanism of action of ferroptosis is crucial for potential applications in OP therapy.

Although numerous clinical agents are effective for restoring bone strength targeting OP, they inadvertently decrease bone strain. Also, some clinical agents are not only costly but also associated with significant adverse effects, requiring long-term use (Li Z. et al., 2023). Natural products possess unique advantages, such as multi-target synergy, low price, low toxicity, and large molecular weight; thus, they hold broad application prospects (Zhang Y. et al., 2024; Xin et al., 2025). This review provides a comprehensive examination of recent research on the characteristics and mechanisms of ferroptosis, its effects on bone marrow mesenchymal stem cells (BMSC), OB, OC, and osteocyte, as well as the therapeutic potential of natural products in treating OP by regulating ferroptosis. The review aims to provide the essential theoretical basis for preventing and treating of OP through the regulation of ferroptosis by natural products.

## 2 Ferroptosis and its characteristics

Dixon et al. first defined the concept of “ferroptosis” as a new type of regulated cell death in 2012 (Dixon et al., 2012). As an iron-dependent mode of cell death, iron is essential for the occurrence of ferroptosis (Feng et al., 2022). Ferroptosis is a newly revealed form of programmed cell death (PCD) that is distinct from other PCD (e.g., autophagy, pyroptosis, and apoptosis) and is marked by the excessive accumulation of iron-dependent lipid peroxidation (LPON) leading to cell death (Fang et al., 2019; Khan et al., 2025). Ferroptosis has unique biological traits, such as excessive accumulation of iron, elevated lipid peroxides (LPO), and decreased expression of glutathione peroxidase 4 (GPX4) (Yang Y. et al., 2022). Morphologically, ferroptosis is featured by shrunken mitochondria, reduced mitochondrial cristae, elevated mitochondrial membrane density, rupture of the outer mitochondrial membrane, and intact cell membranes with normal nuclear morphology (Lai et al., 2022). Biochemically, iron-dependent LPON is a distinctive trait of ferroptosis. Intracellular glutathione (GSH) exhaustion and impaired GPX4 activity prevent LPO from being metabolized by GPX4-mediated reduction reaction, whereas Fe^2+^ oxidizes lipids via the Fenton reaction, triggering reactive oxygen species (ROS) accumulation and consequently promoting ferroptosis (Li J. et al., 2020). In genetic terms, the whole process of ferroptosis is governed by multiple genes that primarily implicated in genetic alterations of iron homeostasis, including *nuclear receptor coactivator 4 (NCOA4)*, LPON-related genes, like *acyl-COA synthetase long-chain family member 4* (*ACSL4*), and antioxidant defenses system-related genes, exemplified by *nuclear factor erythroid 2-related factor 2* (*Nrf2*) (Tang et al., 2021). Ferroptosis impacts immune cells via two distinct approaches (Chen et al., 2021b). First, it affects the population and function of immune cells. Second, immune cells identified ferroptotic cells and subsequently initiated an immune response.

## 3 Mechanism of ferroptosis

Clinical studies indicate that bone loss is a common feature of iron overload diseases (Jeney, 2017). Postmenopausal women also show signs of iron overload, which may be a pivotal factor in the susceptibility of older women to OP (Lu et al., 2015). Evidence indicates that ferroptosis may play a key role in the onset and progression of OP. Ferroptosis has unique hallmarks, including disruption of iron homeostasis, impairment of antioxidant stress capacity, and abnormal LPON (Yang X. et al., 2022), Figure 1.

**FIGURE 1:**
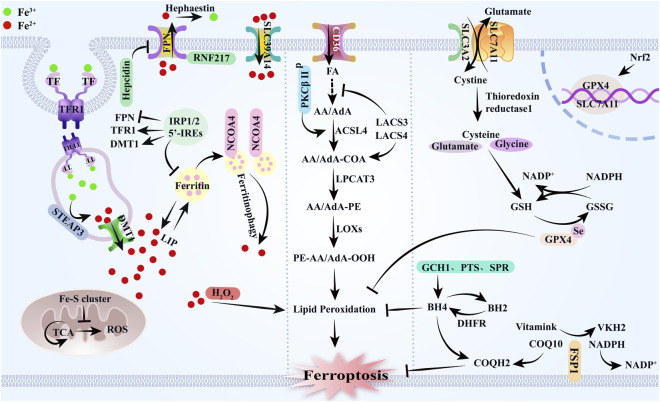
The mechanism and regulation of ferroptosis. The metabolic pathways mediating ferroptosis primarily include iron metabolism, antioxidant regulation, and lipid metabolism. Iron metabolism: Fe^3+^ binds to TF and enters the cell via TFR1. Fe^3+^ entering the cell is reduced to Fe^2+^ by STEAP3 in the endosome, and Fe^2+^ enters the cytoplasm via DMT1. Fe^2+^ can bind to ferritin or be excreted outside the cell via FPN. Hepcidin suppresses FPN to decrease Fe^2+^ release from cells. Ferritin also releases large amounts of Fe^2+^ via ferritinophagy mediated by NACO4. Overall, these processes can increase LIP, thereby sensitizing cells to ferroptosis by the Fenton reaction. Antioxidant regulation: this pathway mainly involves the System Xc-/GSH/GPX4 axis, FSP1/NAD(P)H/CoQ10 axis and GCH1/DHFR/BH4 axis. Cystine is transported into cells via the X_C_- system, promoting GSH synthesis. CoQ10 and vitamin K can be reduced to CoQH2 and VKH2 by FSP1, respectively, inhibiting LPON by scavenging LPON free radicals. GCH1 and DHFR serve as the rate-limiting and catalytic enzymes of BH4, respectively. BH4 can prevent LPON in membranes. Lipid metabolism: AA and AdA, as major PUFA, are metabolized via ACSL4 and LPCAT3, then oxidized by LOXs to promote LPON and ferroptosis. PKCβ II senses initial LPO and boosts the LPON involved in ferroptosis by phosphorylating and activating ACSL4.

### 3.1 Iron metabolism

LPON and toxic ROS derived from iron-driven Fenton reactions and enzymatic oxygenation, which are signature features of ferroptosis (Chen et al., 2022). Cellular iron absorption is primarily mediated by RNA-binding proteins named iron regulatory proteins (IRP), which can regulate the translation of mRNAs encoding transferrin receptor 1 (TFR1), divalent metal transporter 1 (DMT1), ferroportin (FPN), and ferritin light chain (FTL) and heavy chain (FTH) (Dutt et al., 2022). Iron uptake of cells via TFR1 and its release from ferritin-controlled iron pools are critical for sustaining iron balance (Ali et al., 2025). Impaired iron homeostasis participates in the pathogenesis of multiple diseases by initiating the ferroptosis, with transferrin (TF) and its cell surface TFR1 as the critical regulators of ferroptosis (Gao et al., 2015). The intracellular labile iron pool (LIP) is governed by iron uptake, export, storage, and utilization, that in turn are modulated by iron transporters, storage proteins, and regulatory proteins (Wang and Pantopoulos, 2011). Iron mainly exists in two oxidation states within the human body: ferrous iron (Fe^2+^) and ferric iron (Fe^3+^) (Banerjee et al., 2025). Regarding the cellular iron cycle, Fe^3+^ is carried in plasma via binding to TFR1 by TF, and is delivered to diverse tissues and cells. It is subsequently internalized via endosomes and reduced to Fe^2+^ by the enzyme known as six-transmembrane epithelial antigen of the prostate 3 (STEAP3), and ultimately transported into the cytoplasm via DMT1 (Liu X. et al., 2025; Ni et al., 2025). Within the cytoplasm, iron primarily undergoes three key fates (Xie et al., 2025; Xu et al., 2025): (1) After entering the LIP, Fe^2+^ is transferred to mitochondria and other organelles and integrated into iron-containing cofactors such as hemoglobin and Fe-S clusters; (2) to be stored in ferritin, which isolates excess iron and avoids ROS production; and (3) to be exported from the cell via FPN. The Fe^2+^ in LIP are majorly responsible for Fenton reaction-mediated oxidative stress and ferroptosis. The hepatocytes exhibit compensatory upregulation of metal transporter SLC39A14 in the absence of TF expression, resulting in excessive import of iron and thus driving ferroptosis (Jiang et al., 2021). The absorbed Fe^2+^ is either stored within cells by ferritin to avoid the accumulation of excess free iron or carried out of cells via FPN (Liu B. et al., 2025; Zhong et al., 2025). Ferritin consists of a light chain (FTL) and a heavy chain (FTH). FTH possesses ferroxidase activity for the transformation of Fe^2+^ to Fe^3+^, while FTL provides nucleation sites for the formation of mineralized iron cores (Abdukarimov et al., 2025; Huang Q. et al., 2025). Nrf2 regulates the transcription of FTH and FTL to maintain safe intracellular iron storage and prevents excessive accumulation of free Fe^2+^ (Liu K. et al., 2025). Reducing ferritin levels, particularly FTH, elevates the LIP and enhances susceptibility to ferroptosis (Zheng and Conrad, 2020). NCOA4 is a critical protein involved in autophagy-dependent ferritin degradation (Li C. et al., 2025). When iron limitation in the cell, NCOA4 serves as a specific autophagy receptor to mediates the transport of intracellular ferritin to the autophagosome by interacts with FTH1, eventually releasing free iron for systemic physiological requirements (Latunde-Dada, 2017; Shi et al., 2025). The efflux of iron is modulated by FPN, while hepcidin, an important hormone secreted by the liver, can downregulate FPN by E3 ubiquitin-protein ligase ring finger protein 217 (RNF217)-mediated ubiquitination, thus regulating overall iron uptake and circulation (Gong et al., 2025; Ru et al., 2025). The processes of iron storage and release are coordinated by iron-regulatory protein 1/2 (IRP1 and IRP2), which monitor iron levels and regulate iron metabolism-related protein expression by targeting 5′-iron-responsive elements (5′-IREs) (Niu et al., 2025). IRPs enhance intracellular iron uptake and storage under iron-deficient conditions by stabilizing TFR1 and DMT1 mRNA, while simultaneously suppressing FPN and ferritin translation (Jayakumar et al., 2022; Pawłowska et al., 2025).

### 3.2 Lipid peroxidation

Iron metabolism and LPON interact to form the core mechanism of ferroptosis (Wang H. et al., 2025). Excess iron stimulates ROS generation, thereby driving LPON within the cell membranes. The peroxidation of polyunsaturated fatty acid (PUFA)-containing phospholipids in cell membranes serves as a critical step in ferroptosis, whereas LPON primarily targets PUFA (Bayır et al., 2020; Fang et al., 2023). This process can be triggered by two types of reactions: non-enzymatic and enzymatic reactions (Rochette et al., 2022).

The non-enzymatic pathway is facilitated by the Fenton reaction, where iron drives the transformation of hydrogen peroxide into highly reactive hydroxyl radicals (·OH) (Guan et al., 2025). Through stripping hydrogen atoms from the carbon chains of PUFA in the plasma membrane, ·OH trigger a cascade reaction of lipid radicals, resulting in accelerated oxidation and degradation of membrane lipids (Chang et al., 2025; Zhang Y. et al., 2026).

The enzymatic pathway is primarily modulated by enzymes including lipoxygenase (LOX) and cyclooxygenase (COX). Arachidonic acid (AA) and adrenic acid (AdA), derived from PUFA, function as key substrates in the LPON process during ferroptosis (Kagan et al., 2017). Research has identified two membrane remodeling enzymes as critical contributors of LPON in ferroptosis, namely, ACSL4 and lysophosphatidylcholine acyltransferase 3 (LPCAT3) (Gao Z. et al., 2022). ACSL4 initially facilitates the conversion of free AA/AdA to CoA, generating AA/AdA-CoA derivatives and driving their esterification into phospholipids, whereas LPCAT 3 then drives the biosynthesis of AA/AdA-CoA and membrane phosphatidylethanolamine (PE) to generates AA/AdA-PE (Tang et al., 2021; Chen et al., 2025; Patil and Bhatt, 2025). AA/AdA-PE is directly oxidized by lipoxygenases (LOXs), resulting in the LPO formation and ultimately promoting ferroptosis (Sun et al., 2023). Accordingly, the suppression of ACSL 4 or LPCAT 3 diminished ferroptosis in a variety of conditions.

LOXs are a class of non-heme iron-requiring dioxygenase that catalyze the oxidation of PUFAs to generate respective hydroperoxides, and its synthesis can be facilitated by Fe^2+^ in LIP (Kuhn et al., 2015; Zheng et al., 2025b). It is notable that several LOXs are critical in driving ferroptosis, especially arachidonate 5-lipoxygenase (ALOX5), arachidonate 12-lipoxygenase (ALOX12), and arachidonate 15-lipoxygenase (ALOX15). ALOX5 can initiate inflammatory responses and provoke ferroptosis (Sun et al., 2019). Disruption of ALOX12 activity via gene inactivation or missense mutations compromises its ability to catalyze PUFA oxidation and suppresses ROS-driven ferroptosis mediated by p53 (Ru et al., 2025). The complex formed by ALOX15 and phosphatidylethanolamine-binding protein 1 (PEBP1) induces ferroptosis by regulating the substrate specificity of ALOX (Li R. et al., 2025). Latest evidences indicate that membranes regulated the catalytic activity of 15-LOX/PEBP 1 by causing conformational change, which facilitates the formation of peroxidized PUFA-PE and ultimately lead to ferroptosis (Manivarma et al., 2023). PKCβ II senses initial LPO and boosts the LPON involved in ferroptosis by phosphorylating and activating ACSL4 (Zhang et al., 2022; Zeng et al., 2025). LACS4 converts PUFA into acylated forms. It is recognized as a key mediator of ferroptosis because its upregulation elevates the levels of PUFA in phospholipids, making cells more susceptible to ferroptosis (Fang et al., 2023). Conversely, LACS3 facilitates the transformation of exogenous monounsaturated fatty acids into fatty acyl-CoA, thus replacing PUFA and suppressing ferroptosis (Magtanong et al., 2019).

### 3.3 Antioxidant regulation

Ferroptosis can be triggered by two major routes: the exogenous and the endogenous routes (Chen et al., 2021a). The exogenous pathway is initiated either by blocking cell surface transporters proteins (such as the cystine/glutamate transporter, also known as the system Xc^−^) or by activating iron transporters. Conversely, the endogenous pathways are mainly triggered by inhibiting the expression and/or activity of intracellular antioxidative enzymes, including pathways such as the GSH/GPX4 axis and the ferroptosis suppressor protein 1 (FSP1)/coenzyme Q10 (CoQ10) system as well as activating enzymes engaged in fatty acid metabolism such as ACSL4 (Ru et al., 2025).

#### 3.3.1 System X_c_^−^/GSH/GPX4 axis

As an endogenous tripeptide comprised of cysteine, glutamate, and glycine, GSH acts as a key intracellular antioxidant buffer and powerful inhibitor of ferroptosis, guarding cells against oxidative stress-induced damage (Li F. J. et al., 2022; Bai et al., 2025). The reduction of GSH levels impairs GPX4 activity, which impairs the scavenging of LPO and promotes ferroptosis (Wu Q. et al., 2025). GSH can also function as a binding ligand for Fe^2+^ in LIP, preventing the reaction between Fe^2+^ and hydrogen peroxide to generate OH (Huang Q. et al., 2025). The system Xc^−^ transporter is critical for sustaining intracellular GSH levels by serving as an antiporter of cystine/glutamate and enabling cystine import by exchanging it with glutamate (Wang Z. et al., 2024; Hu et al., 2025b). Upon entering the cell, cystine is reduced to cysteine by thioredoxin reductase 1 and subsequently participates in the GSH biosynthesis process mediated by glutamate-cysteine ligase and glutathione synthetase together with glutamate and glycine (Ni et al., 2025). Solute carrier family 7a-member 11 (SLC7A11) is an essential element of the Xc^−^ transporter system and is required for governing cellular uptake of cystine (Liu et al., 2022). Targeted suppression of the system Xc^−^ causes reduced intracellular GSH levels, elevated ROS accumulation, and eventually triggers ferroptosis (Zhou et al., 2020; Lei et al., 2025).

GPX4, a GSH-dependent antioxidant, is an essential hallmark marker of ferroptosis (Yang et al., 2023). GSH delivers necessary electrons to GPX4, enabling the reduction of phospholipid hydroperoxides into nontoxic phospholipid alcohols while simultaneously generating oxidized glutathione (GSSG) (Cao et al., 2025). GSSG is reduced to GSH via GSH reductase in a nicotinamide adenine dinucleotide phosphate (NADPH)-dependent process (Huang C. D. et al., 2025). In response to oxidative stress, Nrf2 upregulates SLC7A11 and GPX4 expression, which consequently prevents ferroptosis (Liu K. et al., 2025). The two classic ferroptosis inducers erastin and RSL3 suppress GPX4 by different processes: erastin depletes GSH through inhibition of system Xc^−^, whereas RSL3 inactivates GPX4 by directly binding to nucleophilic residues of GPX4 (Singh et al., 2025; Xu et al., 2025). Additionally, selenium consumption can directly modulate the antioxidant capability of GPX4 (Lee and Roh, 2025). Despite the normal functioning of the system Xc^−^, selenium deficiency causes a decline in GPX4 activity (Jiang et al., 2025).

#### 3.3.2 FSP1/NAD(P)H/CoQ10 axis

FSP1, a flavin-dependent oxidoreductase, is capable of utilizing NADP(H) to reduce lipophilic free radical scavengers, such as ubiquinone (CoQ10), vitamin K, and various other substrates containing quinones (Tschuck et al., 2025). The function of FSP1 is independent of GSH/GPX4 and can directly scavenge lipid radicals within the plasma membrane and suppress ferroptosis by converting CoQ10 into ubiquinol (CoQ10H2) (Lin et al., 2025; Sun et al., 2025; Yao et al., 2025). Ubiquinol, known as a lipophilic free radical quencher, can directly eliminate lipid radicals and prevents LPON (Ding et al., 2025). Alternatively, it indirectly plays an antioxidant role by recycling α-tocopherol. Dihydroorotate dehydrogenases (DHODH) contribute to antioxidant protection via supporting mitochondrial stability and CoQ10 biosynthesis, thereby further inhibiting ferroptosis (Kureel and Rasmussen, 2025).

Additionally, vitamin K serves as a powerful natural suppressor of ferroptosis, whose function largely hinges on the capacity of FSP1 to effectively convert vitamin K into vitamin K hydroquinone (VKH2). VKH2, as an effective lipophilic antioxidant, inhibited ferroptosis by eliminating oxygen free radicals within the lipid bilayer (Gao et al., 2025; Reznik et al., 2025). In addition to GSH, CoQ10 and vitamin K, functioning as non-enzymatic antioxidants, exert significant antioxidant effects (Liu et al., 2026).

#### 3.3.3 GCH1/DHFR/BH4 axis

Guanosine-5′-triphosphate cyclohydrolase-1 (GCH1)-tetrahydrobiopterin (BH4) axis represents a critical GPX4-independent modulatory pathway in the suppression of ferroptosis. BH4 serves as a cofactor with redox activity, participating in the synthesis of nitric oxide, neurotransmitters and aromatic amino acids (Kraft et al., 2020). BH4 biosynthesis is mediated by a series of enzymatic cascade reactions comprising GCH1, PTS (6-pyruvoyl tetrahydropterin synthase), and SPR (sepiapterin reductase), where GCH1 acts as the rate-limiting enzyme in this pathway (Hu et al., 2022). Meanwhile, dihydrofolate reductase (DHFR) catalyzes the regeneration of BH4 and maintains its anti-ferroptosis function (Soula et al., 2020). BH4 is readily oxidized to dihydrobiopterin (BH2), which forms a redox cycle to suppress ferroptosis by reducing endogenous oxidizing free radicals and protecting lipid membranes (Soula et al., 2020; Wei et al., 2025). Overall, BH4, as a potent endogenous free radical trapping antioxidant, not only modulates GPX4 inhibitor induced ferroptosis susceptibility and protects cells from LPON injury, but also engages in *de novo* synthesis of CoQ10, which also has a protective effect against ferroptosis (Wei et al., 2025).

## 4 Relationship between osteoporotic target cells and ferroptosis

Bone remodeling is a dynamic process involving resorption and deposition phases, mainly driven by OB and OC (Zhang Y. et al., 2024; Wang Y. B. et al., 2025). OB facilitates the formation of new bone, thus maintaining the stability and strength of bone structure. Simultaneously, OC breaks down bone tissue, subsequently delivering minerals and nutrients into the circulatory system (Hu H. T. et al., 2025). Moreover, osteocyte is the predominate cell type in mature bone and is essential for modulating bone turnover (Wu Y. et al., 2025). In the pathological process of OP, it has been discovered that ferroptosis can occur in OB, OC, and osteocyte-all of which are tightly associated with bone metabolism (Wang D. et al., 2025), Figure 2.

**FIGURE 2:**
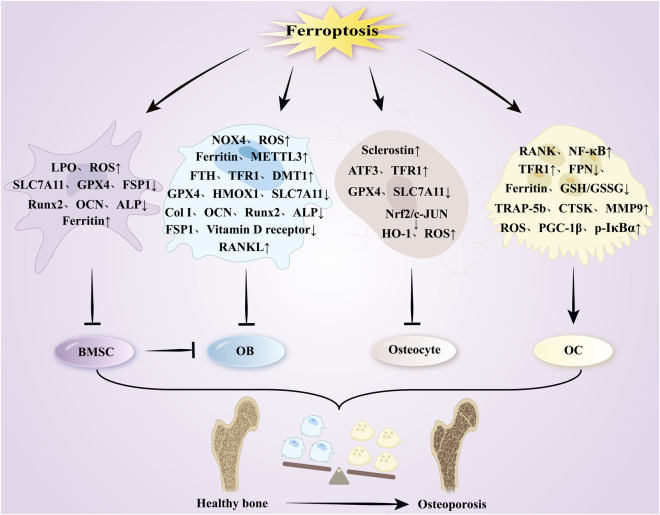
The relationship between OP and ferroptosis in target cells. BMSC upregulate Ferritin expression in response to excess iron, while simultaneously affecting antioxidant regulation. This leads to ROS accumulation, impairing the survival, proliferation, and osteogenic differentiation capacity of BMSC. Similarly, it also affects the function of OB and osteocytes. Upregulation of TFR1 and downregulation of FPN in OC lead to intracellular Fe^2+^ accumulation, enhancing OC differentiation and promoting bone resorption. Ultimately, bone resorption exceeds bone formation, leading to OP.

### 4.1 The negative effects of ferroptosis on bone marrow mesenchymal stem cell

During the maturation and remodeling of bone tissue, BMSC serve as the primary source for OB differentiation (Nan et al., 2025). Iron overload can induce LPON and ferroptosis in BMSC, thereby disrupting bone homeostasis (Yang et al., 2024). The LPON caused by ferroptosis can compromise cell membrane integrity, leading to alterations in the composition and fluidity of BMSC membranes, ultimately inhibiting their differentiation into OB (Xiao W. et al., 2025). There is evidence indicating that ROS exert adverse effects on the survival, proliferation, and terminal differentiation of BMSC (Atashi et al., 2015). It is presently believed that OB are differentiated from BMSC, and that iron can elevate ROS levels in BMSC (Yuan et al., 2019). Excess ROS triggers suppression and apoptosis of BMSC via signaling pathways including Wnt, Hedgehog, and FoxO, thus promoting adipogenesis and depressing differentiation to OB (Atashi et al., 2015). Furthermore, Iron overload upregulated the levels of ferritin in BMSC in a concentration-dependent manner, downregulated the expression of runt-related transcription factor 2 (Runx2), osteocalcin (OCN), and alkaline phosphatase (ALP), and impaired osteogenic differentiation of BMSC (Balogh et al., 2016). In senile osteoporotic BMSC, the expression of SLC7A11, FSP1, and GPX4 is suppressed, while LPO are elevated (Li Z. et al., 2025). Li et al. revealed that the ferroptosis of BMSC was induced by dexamethasone *in vitro*, and that melatonin could ameliorate the situation by activating the PI3K-AKT-mTOR signaling pathway and increasing the levels of GPX4 and FSP1, which was validated in the experiments *in vivo* (Li M. et al., 2022).

### 4.2 The negative effects of ferroptosis on osteoblast

OB is the primary functional cells engaged in bone formation and are involved in the synthesis, secretion, and mineralization of the bone matrix (Xie et al., 2023). OB are among the target cells for iron effects, and various studies have demonstrated that iron suppresses OB function and differentiation (Chen et al., 2024; Sun et al., 2026). Ferroptosis and GPX4 inhibition primarily occurred in OB. ferroptosis in OP primarily involves the following biochemical processes (Wang D. et al., 2025): (1) Excessive Fe^2+^ produces large amounts of ROS and LPO via the Fenton reaction, disrupting the cellular membrane structure of OB; (2) Decreased cysteine uptake and GSH depletion suppress GPX4 activity, resulting in impaired LPO scavenging and compromised OB membrane integrity and osteogenic capacity; (3) FTH and NCOA4 can act synergistically to induce autophagic degradation of ferritin, releasing substantial amounts of Fe^2+^ and triggering ferroptosis. Under conditions of iron overload, OB generate overabundance of ROS, and this activation triggers the RIPK1-RIPK3-MLKL pathway, causing OB ferroptosis and ultimately bone loss (Tian et al., 2020). The excessive iron accumulation facilitates the dissociation of the IRP1 protein from the IRE-like sequences at the *NADPH oxidase 4* (*NOX4*) promoter, which activates *NOX4* transcription. Heightened levels of NOX4 enzyme may trigger enhanced ROS generation and intracellular LPO accumulation, ultimately driving ferroptosis in OB (Zhang H. et al., 2023). Additionally, ROS activate extracellular signal-regulated kinases and heat shock factor 2 to stimulate receptor activator of NF-κB ligand (RANKL) expression (Huo et al., 2024). When the RANKL/OPG ratio is altered, it inhibits osteogenic capacity of OB and stimulates OC differentiation. Doyard et al. found that iron can decrease *collagen type I-alpha1* chain, *OCN*, and *RUNX2* mRNA expression levels by reduction of *Hedgehog interacting protein like-2* mRNA expression levels, which ultimately reduces OB activity (Doyard et al., 2012). In the zebrafish model, iron overload increased ROS levels and also decreased the levels of OB-specific genes, such as *type I collagen* (*COL I*), *ALP* and *RUNX2*, leading to decreased bone formation (Chen et al., 2014). Luo et al. performed *in vitro* studies using ferric ammonium citrate (FAC) to induce ferroptosis in OB and revealed that it upregulates the cellular expression of *TFR1* and *DMT1* genes, which enhanced intracellular iron uptake and subsequently triggered the generation of ROS and LPO, eventually leading to ferroptosis (Luo et al., 2022). Ferroptosis inhibitors may safeguard OB and sustain their differentiation by reactivating the canonical Wnt signaling pathway. Jiang et al. indicated that iron overload triggers OB ferroptosis by suppressing GPX4, HMOX1, and SLC7A11 expression while elevating FTH expression, which consequently impairs OB differentiation and mineralization (Jiang et al., 2022). Lin et al. established the diabetic OP model and found that circulating ferritin levels and serum iron levels were significantly elevated, and SLC7A11 and GPX4 expression were decreased (Lin et al., 2022). Meanwhile, OB cell lines subjected to high glucose and palmitic acid treatment exhibited impaired ability in osteogenic differentiation and mineralization, and suffered ferroptosis. Knockdown of methyltransferase-like 3 (METTL3), a major m6A methyltransferase, can inhibit OB ferroptosis by blocking the METTL3/ASK1-P38 signaling pathway. Xu et al. confirmed that activation of vitamin D receptors can attenuate ferroptosis in OB by triggering the Nrf2/GPX4 signaling pathway, thus preventing age-related OP (Xu et al., 2022). Tong et al. observed that inhibiting FSP1 promotes OB ferroptosis in the D-galactose-induced OP *in vitro* model (Tong et al., 2025). Other studies have confirmed that iron overload suppresses OB proliferation and mineralization by downregulating the PI3K/AKT/FOX3a/DUSP14 pathway, and compromises OB differentiation by arresting the cell cycle in the proliferative G1 phase (Cen et al., 2018; Xia et al., 2019).

### 4.3 The positive effects of ferroptosis on osteoclast

OC is generated from the fusion of mononuclear lineage precursor cells and releases acidic substances and proteases to dissolve and absorb old bone (Liu C. et al., 2025). Increased OC differentiation and bone resorption are regarded as the primary reasons for iron overload leading to bone loss (Che et al., 2020). During OC differentiation, there is an elevated requirement for iron and consequent upregulation of TFR1 expression, thus enhancing cellular iron uptake (Ni et al., 2021; Gao L. et al., 2022). Conversely, decreased FPN expression promotes OC differentiation during the initial stages of OC maturation (Xiao R. et al., 2025). Enhanced iron absorption and PGC-1β exhibit a synergistic effect, thereby increasing both the quantity and functionality of mitochondria in OC. Increased mitochondria resulted in more ROS, which enhanced PGC-1β expression and stimulated OC differentiation (Ishii et al., 2009). The ROS produced by excess iron serve as the ultimate executors of ferroptosis and also act as potent activators of signaling pathways that promote OC differentiation and resorptive activity, such as NF-κB (Motafeghi et al., 2026). Aberrant IRPs in OC also disrupt iron metabolism, impair mitochondrial respiratory chain, and produce more ROS (Wang J. et al., 2024). Wang et al. confirmed that iron accumulation contributes to elevated ROS levels, which then stimulate OC differentiation by the NF-κB signaling pathway (Wang et al., 2018). Research reveals that OPG level impacts the redox coupling of GSH/GSSG, ultimately influencing OC formation, while the ratio of OPG to GSH exhibits a negative correlation. Interestingly, a physiologically reduced GSH/GSSG ratio was confirmed to promote OC formation during the terminal stage of OC differentiation (Hou et al., 2023). RANKL is secreted by OB and interacts with RANK on the surface of OC precursor cells. This interaction not only stimulates the differentiation of OC precursor cells into OC but also suppresses OC apoptosis (Wang et al., 2022). Research has indicated that excess iron enhances both the expression of RANKL and the differentiation of OC (Chen H. et al., 2023). Ferroptosis can be triggered by combining RANKL stimulation with an iron starvation response characterized by upregulation of TFR1 expression and downregulation of ferritin (Yang H. et al., 2025). The intraperitoneal injection of iron citrate in ovariectomized mice significantly elevated ROS generation within OC and promoted the upregulation of RANKL-induced genes such as *TRAP-5b*, *CTSK*, and *MMP9*. This process is accompanied by elevated expression of phosphorylated IκBα and stimulation of NF-κB signaling pathway, thereby accelerating OC differentiation and enhancing bone resorption activity, ultimately causing aggravated bone loss in mice (Xiao et al., 2015). Therefore, ferroptosis may facilitate osteoclastogenesis by ROS-dependent mechanisms and RANKL-mediated pathways.

### 4.4 The negative effects of ferroptosis on osteocyte

Osteocyte, as terminally differentiated OB, are responsible for communicating with their surrounding environment after embedding into the mineralized bone matrix and can influence OB and OC by releasing signaling molecules (Palumbo and Ferretti, 2021; Wu et al., 2024). As the predominant cell type in bone tissue, osteocyte possesses the ability to detect mechanical stress and regulate bone formation and resorption (Zhu et al., 2026). Yang et al. revealed that iron excess triggers apoptosis and promotes RANKL secretion in MLO-Y4 osteocyte-like cells, which subsequently drives the osteoclastogenic differentiation of co-cultured monocytes (Yang et al., 2020). Ma et al. demonstrated that iron excess accelerates osteocyte apoptosis and bone mass loss via the upregulation of sclerostin and the imbalance of RANKL/OPG using a hepcidin knockout mouse model (Ma et al., 2022). Yang et al. demonstrated that the microenvironment of type 2 diabetes facilitates *HO-1* transcription and elevates ROS levels via the Nrf2/c-Jun signaling axis, while concurrently suppressing GPX4 expression (Yang Y. et al., 2022). This dual regulatory mechanism drives osteocyte ferroptosis, thereby promoting the progression of diabetic OP. Chankamngoen et al. revealed that iron excess triggers apoptosis in osteocyte and upregulates the *SOST* gene (encoding sclerostin) and the *Tnfsf11* gene (encoding RANKL) levels using several different osteocyte models *in vitro* (Chankamngoen et al., 2023). In addition, it was found that iron exposure stimulates the upregulation of fibroblast growth factor 23, which also facilitates the differentiation and survival of osteoclast-like cells. Guo et al. reported that iron excess triggers osteoclastogenesis via oxidative stress by stimulating osteocyte apoptosis and RANKL generation (Guo et al., 2024). Yin et al. discovered that activating transcription factor 3 (ATF3) acts as a key regulator in osteocyte ferroptosis via single-cell sequencing (Yin et al., 2024). Elevated ATF3 expression in senescent osteocyte not only upregulated TFR1 to promote iron uptake, but also inhibited cystine import mediated by SLC7A11, which ultimately led to iron overload and loss of cortical bone mass. Jiang et al. demonstrated that osteocyte in ovariectomized mice undergo ferroptosis and that RANKL expression levels are upregulated in this process, resulting in overactivation of OC and ultimately OP (Jiang Z. et al., 2024). Furthermore, this research also revealed that inhibition of Nrf2 resulted in the downregulation of DNA methyltransferase 3a (DNMT3a)-mediated methylation levels of the RANKL promoter, which is a crucial mechanism for osteocytic ferroptosis promoting osteoclastogenesis. Iron overload has been proven to not only disrupt the microfilament skeleton of osteocyte but also cause alterations in the distribution of the microfilament skeleton in osteocyte (Wang D. et al., 2025). Osteocytic dendritic processes across the lacunar-canalicular system regulate the mechanosensitivity of SLC7A11, modulating redox capacity in response to mechanical stimulation (Yang D. et al., 2025).

## 5 The therapeutic potential of natural products on OP through ferroptosis

Natural products are widely recognized for their greater safety profile compared to synthetic drugs and exhibit growing potential in the treatment of OP (Yang X. et al., 2025). Since the concept of ferroptosis was proposed, numerous natural products have been demonstrated to exert anti-osteoporotic effects by regulating ferroptosis (Yang et al., 2023). It is well known that the essence of OP involves an imbalance between OB and OC. Hence, we reviewed how natural products exert anti-osteoporotic effects by regulating ferroptosis Table 1.

**TABLE 1:** Natural products for the prevention and treatment of OP by ferroptosis.

Natural products	Extract type/Solvent	Optimal dose	Mechanisms related to ferroptosis regulation	Affecting cells	Clinical studies	References
Sarsasapogenin	Purified monomer	0.1/1 μM (*in vitro*); 5/10 mg/kg/d, ig, 12 weeks (*in vivo*)	Upregulating GPX4 expression and stimulating type-H blood vessels formation	BMSC and endothelial cells	No	Wang F. et al. (2025)
Poliumoside	Purified monomer	40 μM (*in vitro*); 7.5/15 mg/kg, ip, 8 weeks (*in vivo*)	Increasing GPX4 expression and enhancing the synthesis of osteogenesis-related proteins	BMSC	No	Xu et al. (2024)
Astragaloside IV	Purified monomer	25/50/100/200 μM (*in vitro*); 40 mg/kg/2d, ip, 4 weeks (*in vivo*)	Suppressing ferritin and FPN1 expression and increasing TFR1 expression	BMSC	No	Jin et al. (2021)
Astragalus Polysaccharide	Purified monomer	10/30/100 μg/mL (*in vitro*)	Reduction of mitochondrial ROS accumulation	BMSC	No	Yang et al. (2016)
Quercetin	Purified monomer	0.03 μM (*in vitro*)	Reduction of ROS accumulation	BMSC	Yes	Li X. et al. (2020)
Picein	Purified monomer	80 μM (*in vitro*); 40/80 μM/d, Femoral condyle injection, 4 weeks (*in vivo*)	Activating the Nrf2/HO-1/GPX4 axis to stimulate osteogenic differentiation of BMSC	BMSC	No	Huang et al. (2024)
Aucubin	Purified monomer	5/10 μM (*in vitro*); 30 mg/kg/2d, ip, 8 weeks (*in vivo*)	Upregulating SLC7A11 and SLC3A2 expression and activating the BMP2/SMAD pathway	BMSC	No	Zheng et al. (2025a)
Forsythiaside A	Purified monomer	80 μM (*in vitro*); 80 mg/kg, 8 weeks (*in vivo*)	Facilitating nuclear translocation of Nrf2 and elevating expression of GPX4	BMSC	No	Huang Y. X. et al. (2025)
Salidroside	Purified monomer	80 μM (*in vitro*); 30/60 mg/kg/day, ig, 12 weeks (*in vivo*)	Activating the PI3K/AKT/mTOR pathway while elevating the expression of SLC7A11, GPX4 and FSP1, and reducing the expression of ACSL4	BMSC	No	Li Z. et al. (2025)
Leonurine	Purified monomer	10 μM (*in vitro*); 15 mg/kg/day, ip, 2 months (*in vivo*)	Activating the JNK signaling pathway to suppress ferroptosis in BMSC and promoting M2 polarization to inhibit OC differentiation	BMSC and OC	No	Lan et al. (2026)
Asperosaponin VI	Purified monomer	50 mg/kg, three times per week, ip, 6 weeks (*in vivo*)	Enhancing the expression of GPX4	OB	Yes	Wei et al. (2024)
Mangiferin	Purified monomer	0.001/0.01/0.1 μM (*in vitro*); 10/50/100 mg/kg/d, ig, 12 weeks (*in vivo*)	Elevating Nrf2, GPX4 and SLC7A11 expression	OB	No	Deng et al. (2024)
Icariin	Purified monomer	0.01/0.1/1 μM (*in vitro*); 100 mg/kg/day, ig, 2 months (*in vivo*)	Preventing MMP dysfunction and ROS generation and upregulating Runx2, ALP, and OCN expression	OB	Yes	Jing et al. (2019)
Silymarin	Purified monomer	50 mM (*in vitro*); 50 mg/kg/day, ip, 12 weeks (*in vivo*)	Promoting the expression of Runx2, Sirtuin 1, OCN and superoxide dismutase 2	OB	No	Tao et al. (2022)
Curculigoside	Purified monomer	10 μM (*in vitro*); 25/50/100 mg/kg/day, ig, 3 months (*in vivo*)	Suppressed the activation of IGFR/AKT signaling pathway and upregulated the expression levels of FoxO1 and Nrf2	OB	No	Zhang et al. (2019)
Resveratrol	Purified monomer	2/10/50 μM (*in vitro*); 30/60/90 mg/kg/day, ig, 3 months (*in vivo*)	Upregulating the expression of FoxO1 and reversing the reduction of Runx2, OCN and COLI	OB	Yes	Zhao et al. (2015)
Baicalein	Purified monomer	1/10 μM (*in vitro*); 10/100 mg/kg/day, ig, 8 weeks (*in vivo*)	Activating the Nrf2/GPX4 Pathway	OB	Yes	Guo et al. (2026)
Syringaresinol	Purified monomer	25/50/100 μM (*in vitro*)	Activating the Nrf2/SLC7A11/GPX4 pathway	OB	No	Wang and Zhang (2026)
Proanthocyanidins	Purified monomer	1 μM (*in vitro*); 10/20 mg/kg, injection, 8 weeks (*in vivo*)	Activating the SIRT6/Nrf2/GPX4 pathway	OB	Yes	Ma et al. (2025)
Picroside II	Purified monomer	50 μM (*in vitro*); 10/20/40 mg/kg/day, ig, 8 weeks (*in vivo*)	Upregulating GPX4 and SLC7A11 expression	OB	No	Wang and Qian (2025)
Qing’e pill	Hot water extraction + ethanol (95%) reflux extraction	20 mg/mL (*in vitro*); 4.5 g/kg/day, ig, 2 weeks (*in vivo*)	Inhibiting ATM serine/threonine kinase and promoting the PI3K/AKT pathway	OB	Yes	Hao et al. (2022)
Fructus Ligustri Lucidi	—	1.56 g/kg/day, ig, 12 weeks (*in vivo*)	upregulating GPX4 and SLC7A11 expression by activating the Nrf2/HO-1 pathway	OB	Yes	Li P. et al. (2023)
Artemisiae Scopariae Herba	Water solution	5/10 g/kg/day, ig, 12 weeks (*in vivo*)	Stimulating the Nrf2/SLC7A11/GPX4 pathway	OB	No	Li et al. (2026)
Yishen Gushu formula	Hot water extraction	5% drug-containing serum (*in vitro*); 1.944 g/mL, ig, 12 weeks (*in vivo*)	Downregulating NCOA4 expression and upregulating SLC7A11, GPX4, and FTH1 expression	OB	Yes	Jin et al. (2026)
Psoraleae Fructus combined with Walnut kernels	N-hexane, ethyl acetate, 95% ethanol, and ultrapure water reflux extraction	NA: 5 μM + ALA: 0.5 μM (*in vitro*); 1.0/4.0 g/kg/day, 8 weeks (*in vivo*)	Stimulating Nrf2 nuclear translocation to upregulate downstream HO-1, GPX4, and SLC7A11 expression while repressing TFR1-mediated iron overload	OB	No	Hu et al. (2025c)
Aconine	Purified monomer	10/20 μM (*in vitro*); 5 mg/kg/day, ip, 8 weeks (*in vivo*)	boosting GPX4 expression and suppressing ACSL4 expression	OC	No	Xue et al. (2023)
Zingerone	Purified monomer	400 μM (*in vitro*); concentration: 2 mg/mL, 1.25 mL/25 g/day, ig, 8 weeks (*in vivo*)	Elevated ferroptosis sensitivity by p53-mediated regulation of SAT1 and GPX4 expression	OC	No	Li H. et al. (2025)
Hops extract and Xanthohumol	Ethanol (75%) reflux extraction	Hops extract: 1.5/3/6 μg/mL (*in vitro*); 1/3 g/kg/day, ig, 12 weeks (*in vivo*) and Xanthohumol: 1.25/2.5/5 μM (*in vitro*); 30/90 mg/kg/day, ig, 12 weeks (*in vivo*)	Activation of AKT/GSK3β/Nrf2 pathway	OB and OC	Yes	Sun et al. (2022)

### 5.1 Natural products ameliorate OP by regulating ferroptosis in bone marrow mesenchymal stem cell

Sarsasapogenin not only promotes the osteogenic activity of BMSC by suppressing ferroptosis in a GPX4-dependent manner, but also promotes type-H blood vessels formation by increasing the level of slit guidance ligand 3 derived from BMSC and activating roundabout human homologous-1 receptors in endothelial cells, ultimately mitigating bone loss by enhancing the coupling between osteogenesis and angiogenesis (Wang F. et al., 2025). Sarsasapogenin was confirmed to bind directly to GPX4 and potentially enhance its functional activity through cellular thermal shift assay surface plasmon resonance (SPR), and molecular docking (MD). Sarsasapogenin exhibits mostly uniform and unspecific distribution within the body, which may lead to side effects and reduced bioavailability in target tissues, thereby hindering clinical translation (Mustafa et al., 2022). Poliumoside enhances Nrf2 nuclear movement, promotes GPX4 expression and augments synthesis of osteogenesis-related proteins (such as Runx2, OCN, and COL1A1), which promotes BMSC differentiation and mineralization and ameliorates bone loss in DOP mice (Xu et al., 2024). Poliumoside was confirmed to bind directly to Nrf2 and GPX4 by MD. Poliumoside is easily hydrolyzed and metabolized into degradation products *in vivo*, which also contributes to its low bioavailability (Deng et al., 2014). Astragaloside IV can regulate iron homeostasis and metabolism by inhibiting ferritin and FPN1 expression and increasing TFR1 expression in BMSC, thereby inhibiting iron overload caused by FAC (Jin et al., 2021). The efficacy of Astragaloside IV still needs to be confirmed by clinical trials, and its bioavailability under different dosage and administration routes in different animal models has not yet been fully elucidated (Yao et al., 2023). Astragalus Polysaccharide significantly attenuated the mitochondrial ROS accumulation in BMSC, which protects BMSC from ferroptosis, and ultimately restores their proliferation and differentiation capabilities (Yang et al., 2016). Limited research on the structural characteristics of Astragalus Polysaccharide may be a critical constraint on clinical studies. Therefore, conducting more clinical research based on sufficient structural characterization studies could represent an important future research direction (Li C. X. et al., 2022). Similarly, Quercetin markedly decreased the accumulation of ROS and prevented erastin induced ferroptosis in BMSC (Li X. et al., 2020). Although several clinical trials have been conducted based on the pharmacological effects of Quercetin, its clinical translation remains limited due to its low bioavailability and considerable variations among individuals (Devi et al., 2024). Picein can alleviate oxidative stress damage by activating the Nrf2/HO-1/GPX4 pathway, ultimately preventing erastin induced ferroptosis in BMSC. Additionally, Picein also promotes macrophage M2 polarization (Huang et al., 2024). Picein is active component of various herbs and can be extracted from different types of herbs, making it more readily available (Elyasi et al., 2022). Picein possesses a structural similarity of 92% to gastrodin and exhibits potent antioxidant activity (Kesari et al., 2025). Aucubin can antagonize erastin-induced ferroptosis in BMSC by upregulating SLC7A11 and SLC3A2 expression, suppressing ROS and intracellular Fe^2+^ levels, and promoting GPX activity. It further enhances osteogenic differentiation by activating the BMP2/SMAD pathway (Zheng et al., 2025a). Pharmacokinetic studies indicate that intraperitoneal administration of Aucubin provides higher bioavailability compared to oral administration. This may be due to the instability of Aucubin in acidic gastric juice, poor gastrointestinal absorption caused by its low lipophilicity, and possible first-pass effect in the liver (Kartini et al., 2023). Forsythiaside A may suppress ferroptosis in BMSC by facilitating Nrf2 nuclear translocation and elevating GPX4 expression, thereby alleviating the inhibitory effects of high glucose and high fat on BMSC osteogenic differentiation and mineralization (Huang Y. X. et al., 2025). MD confirmed that Forsythiaside A can bind to Nrf2 and GPX4. Pharmacokinetic research indicates that Forsythiaside A exhibits low absorption and bioavailability and is rapidly eliminated *in vivo* (Gong et al., 2021). Salidroside remarkably suppress ferroptosis in BMSC by stimulating the PI3K/AKT/mTOR pathway, particularly via elevating the expression of SLC7A11, GPX4, and FSP1 while reducing ACSL4 expression, thereby alleviating OP (Li Z. et al., 2025). Despite its excellent safety and tolerability, Salidroside has limited bioavailability in animals and is rapidly hydrolyzed and conjugated (Chaitanya et al., 2025). Leonurine can inhibit ferroptosis in BMSC by activating the JNK signaling pathway, thereby promoting their osteogenic function. Meanwhile, Leonurine also suppresses OC differentiation by facilitating M2 polarization (Lan et al., 2026). MD confirmed that Leonurine can bind to JNK. Leonurine also suffers from low bioavailability and high first-pass elimination, and its safety profiling and toxicological research require further reinforcement (Liu S. et al., 2024).

### 5.2 Natural products alleviate OP by regulating ferroptosis in osteoblast

Asperosaponin VI alleviates the downregulation of GPX4 via suppressing the abnormal upregulation of DNMT1/3a and the hypermethylation of the GPX4 promoter, ultimately suppressing ferroptosis in OB and ameliorating bone loss in DOP mice (Wei et al., 2024). Direct interactions between Asperosaponin VI and DNMT1/3a have been confirmed by pull-down assays and MD. Despite its low bioavailability in rats, Asperosaponin VI has been successfully developed as a novel drug to treat OP (Wang et al., 2015; Ding et al., 2019). Mangiferin, functions as a direct inhibitor of Keap1, facilitates its degradation by interacting with Keap1, while stimulating Nrf2 to initiate the downstream SLC7A11/GPX4 signaling, thereby suppressing OB ferroptosis (Deng et al., 2024). Mangiferin can bind directly to the Keap1, thus potentially promoting the release and activation of Nrf2, which has been confirmed by SPR and MD. Despite exhibiting promising pharmacological effects in multiple preclinical studies, the clinical translation of Mangiferin has been limited by its poor solubility, absorption, and overall bioavailability (Shang et al., 2025). Icariin can prevent mitochondrial membrane potential (MMP) dysfunction and ROS overproduction, and reverse the downregulation of Runx2, ALP, and OCN expression caused by iron excess, thereby protecting OB from ferroptosis (Jing et al., 2019). Clinical trials have demonstrated that Icariin can effectively delay the onset of OP with good tolerability and almost no adverse reactions. However, it similarly faces the challenge of poor oral bioavailability, which limits its clinical application (Yuan et al., 2022). Silymarin enhances the expression of Runx2, Sirtuin 1, OCN and superoxide dismutase 2 in the iron overload environment and inhibits OB ferroptosis, which promotes the proliferation and differentiation of OB (Tao et al., 2022). As a potent antioxidant, Silymarin has shown good tolerability and almost no adverse reactions in clinical trials conducted to date. (Gillessen and Schmidt, 2020). Current preclinical studies suggest that Silymarin may exert anti-osteoporotic effects by regulating redox homeostasis, but further research is needed to confirm its clinical efficacy. Curculigoside could mitigate the impact of excessive iron on OB proliferation and differentiation potential by inhibiting the IGFR/AKT signaling pathway, upregulating the expression levels of forkhead box protein O1 (FoxO1) and Nrf2, suppressing FoxO1 phosphorylation, and facilitating FoxO1 nuclear translocation (Zhang et al., 2019). The poor oral bioavailability of Curculigoside may be caused by P-glycoprotein mediated efflux and high intrinsic clearance in rat liver, hindering its clinical translation (Wang et al., 2016). Additionally, Resveratrol has been proven to upregulate the expression of FoxO1 and reverse the downregulation of Runx2, OCN, and COLI induced by iron overload, thereby alleviating ferroptosis in OB (Zhao et al., 2015). Although multiple clinical trials on Resveratrol have been conducted, it still faces challenges such as rapid metabolism and poor systemic bioavailability. Furthermore, large-scale and long-term clinical trials are required to validate its therapeutic efficacy (Brown et al., 2024). Baicalin functions as both an iron chelator and an antioxidant. Baicalin may promote bone formation by reversing the decrease in GSH/GSSG ratio and accumulation of LPO caused by iron overload, which is achieved by increasing Nrf2 levels in OB nuclei and the expression of downstream targets GPX4 and SLC7A11 (Guo et al., 2026). Baicalin has completed human safety and pharmacokinetic trials, indicating no significant side effects even at high doses (Li et al., 2021). Syringaresinol can activate the Nrf2/SLC7A11/GPX4 pathway to promote FSP1 expression and suppress ACSL4 expression, thereby reducing cytoplasmic free iron levels and simultaneously decreasing ROS and MDA levels, ultimately alleviating OB ferroptosis. Additionally, rats administered Syringaresinol orally exhibit slow absorption, extensive distribution, and high safety (Wang and Zhang, 2026). Proanthocyanidins can reverse high glucose induced OB ferroptosis by activating the SIRT6/Nrf2/GPX4 pathway, thereby alleviating diabetic OP (Ma et al., 2025). MD confirmed that Proanthocyanidins can bind to SIRT6. Although Proanthocyanidins with higher molecular weight exhibits lower bioavailability, they possess greater gastric stability and higher potential scavenging activity (Izumi and Terauchi, 2020). Picroside II may inhibit OB ferroptosis by upregulating GPX4 and SLC7A11 expression, thereby regulating the Yin Yang 1/transforming growth factor-β1 axis and ultimately ameliorating OP (Wang and Qian, 2025). The poor gastrointestinal absorption of Picroside II due to their hydrophilic nature is the primary cause of their low bioavailability (Wu et al., 2023).

Qing’e pill suppresses ferroptosis and promotes OB differentiation by inhibiting ATM serine/threonine kinase and promoting the PI3K/AKT pathway (Hao et al., 2022). In clinical practice, Qing’e pill has been widely used to treat OP-related conditions, including postmenopausal OP and diabetes-induced OP (Wang Y. et al., 2025). Fructus Ligustri Lucidi has been clinically used to manage osteoporotic bone pain and rheumatic bone for more than 1,000 years (Chen et al., 2017). Fructus Ligustri Lucidi could upregulate GPX4 and SLC7A11 expression levels by activating the Nrf2/HO-1 signaling pathway, which subsequently suppressed ferroptosis and LPO levels during OP (Li P. et al., 2023). Artemisiae Scopariae Herba may alleviate OP by stimulating the Nrf2/SLC7A11/GPX4 pathway, thereby suppressing OB ferroptosis, elevating OPG, RUNX2, and GSH levels, and reducing TRAP, MDA, and 4-HNE levels (Li et al., 2026). Traditional Chinese medicine formulations based on Artemisiae Scopariae Herba have been developed into chinese patent medicine and marketed, demonstrating excellent safety and efficacy (Wei et al., 2022). However, no clinical trials have yet been conducted on the use of Artemisiae Scopariae Herba for the treatment of OP. Yishen Gushu formula may reverse OB ferroptosis by downregulating NCOA4 expression and upregulating SLC7A11, GPX4, and FTH1 expression, thus ameliorating OP. Clinical trials have proven that Yishen Gushu formula can effectively enhance BMD in patients via modulating the bone formation-resorption coupling mechanism (Jin et al., 2026). Psoraleae Fructus combined with Walnut kernels can reverse osteogenic dysfunction by stimulating Nrf2 nuclear translocation to upregulate downstream HO-1, GPX4, and SLC7A11 expression while repressing TFR1-mediated iron overload. Further research identifies norbakuchinic acid (NA) and α-linolenic acid (ALA) as the primary bioactive components (Hu et al., 2025c). Both NA and ALA can bind directly to Keap1, facilitating Nrf2 nuclear translocation and triggering downstream biological responses, which has been confirmed by MD, molecular dynamics simulations, and microscale thermophoresis.

### 5.3 Natural products mitigate OP by regulating ferroptosis in osteoclast

Aconine could upregulate GPX4 expression and downregulate ACSL4 expression in OC by blocking the activation of the NF-κB signaling pathway, which reduced the numbers and activity of OC (Xue et al., 2023). Zingerone can upregulate SAT1 protein expression and downregulate GPX4 protein expression by increasing p53 protein expression, thereby elevating ROS and Fe^2+^ levels and ultimately enhancing the sensitivity of OC precursor cells to ferroptosis, reversing bone loss in OP (Li H. et al., 2025). The strong binding affinity between the Zingerone and p53 has been confirmed by MD. Zingerone have the features of rapid absorption and high bioavailability *in vivo*, with relatively low toxicity and no off-target effects (Rashpa et al., 2025).

Hop extract and Xanthohumol can activate the AKT/GSK3β/Nrf2 pathway to enhance the antioxidant level, which regulates the level of parathyroid hormone to facilitate the transformation of VD3, thereby indirectly promoting the intestinal absorption of calcium, balancing the differentiation of OB and OC, and effectively ameliorating the bone loss and improving the bone microstructure in the iron-overloaded mice (Sun et al., 2022). A clinical trial has demonstrated that Hop extract may protect postmenopausal individuals from OP, with no significant adverse reactions reported (Kim et al., 2021).

## 6 Natural product nano-delivery systems for bone

Although natural products are a safer and more economical option, their use remains limited compared to synthetic drugs due to poor bioavailability and metabolic enzymes that restrict their delivery to specific sites (Gupta et al., 2023). Accordingly, effective delivery systems, such as nanomaterials, are needed to address therapeutic challenges associated with natural products, including poor bioavailability, low solubility, poor permeability, first-pass metabolism, and instability in biological environments.

### 6.1 Lipid-based nanomaterials

Lipid-based nanodelivery systems include various formulations, such as liposomes, lipid nanoparticles, and solid lipid nanoparticles. Since liposomes are nanoscale vesicles with a bilayer lipid structure, water-soluble bioactive compounds from plants are encapsulated within their hydrophilic core, while lipophilic compounds are loaded into their hydrophobic lipid bilayer (Zhang S. et al., 2026). Given their intrinsic lipophilicity, lipid-based nanomaterials function as ideal carriers for poorly soluble drugs, significantly enhancing solubility and bioavailability (Hsu et al., 2024). Lipid-based nanomaterials offer exceptional control over size and surface properties, facilitating precise drug delivery tailored to specific pathological mechanisms or tissue types. Encapsulating plant compounds in lipids can also enhance their penetration, absorption, and distribution within the bone tissue (Gilani et al., 2021).

### 6.2 Polymer-based nanomaterials

Polymer-based nanoformulations are composed of natural or synthetic polymers, including micelles, dendrimers, nanogels, and nanocapsules. These types of nanoformulations possess advantages such as cost-effectiveness, improved preparation methods, biocompatibility, and biodegradability (Deljavan Ghodrati et al., 2026). Various methods (such as solvent evaporation, emulsification, and nano-precipitation) can be applied to load plant bioactive compounds within polymer matrices.

### 6.3 Inorganic nanoformulations

Inorganic nanomaterials can be prepared from materials such as silica, and metals or their oxides (Saweres-Argüelles et al., 2023). Inorganic nanomaterials are favored for their high bioavailability, ease of synthesis, functionalization, and sustained release of loaded compounds (Burduşel et al., 2022). Inorganic nanoformulations specifically combine various plant bioactive compounds by directly utilizing these compounds to biologically reduce inorganic materials. During this process, the bioactive components from plants lead to the functionalization of the surface of the nanoscale inorganic materials (Gupta et al., 2023).

In addition, nanofibers, scaffolds with nanocrystals, nanogels, and nanopowders can be effectively used for the controlled delivery of bioactive plant compounds.

## 7 Conclusion

Ferroptosis, a newly identified type of PCD, has drawn substantial scientific interest owing to its potential involvement in the pathogenesis of diverse pathological conditions. Considerable advances have been achieved in relevant studies in recent years, particularly in elucidating the connection between ferroptosis and OP. Investigating the fundamental mechanisms of ferroptosis in OP may provide critical insights into the pathogenesis of OP and offer novel therapeutic approaches for its treatment.

The present review discusses the key features of ferroptosis and its modulatory mechanisms, along with its effects in OP. Moreover, we discussed the beneficial effects of natural products in alleviating OP by regulating ferroptosis via *in vivo* and *in vitro* studies. However, the present research still has many gaps. Firstly, Iron is also an indispensable element for normal cells. How to precisely target ferroptosis in bone-related diseases while mitigating potential damage to normal cells and organs remains a challenge for future research. For example, systemic inhibition of ferroptosis or iron chelation may potentially cause anemia or impair immune cell function. Further, what are the damage thresholds required for iron overload to induce ferroptosis? Iron overload can induce ferroptotic cell death only when it exceeds a certain threshold. Secondly, the onset and advancement of OP hinge on the interplay among various cellular populations, encompassing BMSC, OB, OC, and osteocyte. Nevertheless, the precise nature of whether key ferroptosis regulators-including GPX4, SLC7A11, and Nrf2-display comparable or divergent expression patterns across distinct cellular subtypes within bone tissue during pathological states remains an unresolved scientific question. Furthermore, it is still not fully clarified the functions and downstream mechanisms of these ferroptosis regulators among different cell types in bone tissue. Thirdly, despite remarkable distinctions between ferroptosis and other PCD, emerging evidence underscores functional interplay between ferroptosis and other PCD, including apoptosis and autophagy. For instance, SLC7A11, GPX4, and Nrf2 are not only “core regulators” of ferroptosis but also engage in other PCD. Fourthly, although many natural products have demonstrated anti-osteoporotic effects by regulating ferroptosis at the cellular or animal level, few drugs derived from natural products have been clinically converted for the treatment of OP. On the one hand, screening and verifying natural products that can effectively treat OP in clinical trials based on laboratory data requires considerable workload, human resources, economic expenses, and a significant amount of time. On the other hand, natural products possess drawbacks such as rapid metabolism, poor absorption, low bioavailability, and low specificity. Additionally, limitations in the collection and safety of natural products also hinder its conversion into clinical drugs. Fifthly, sex differences might influence ferroptosis-mediated OP. It is well known that estradiol deficiency primarily promotes bone resorption, while testosterone deficiency inhibits bone formation. In younger men, the beneficial effects of testosterone slightly exceed the inhibitory effects of iron on bone formation; in older men, the opposite occurs, leading to a slow rate of bone loss. In women, the gap between iron and estradiol after the age of 45 is significantly greater than the gap between iron and testosterone in men at the same age. Iron-induced reduction in bone formation, coupled with increased bone resorption due to estradiol deficiency, can accelerate bone loss. Additionally, elderly individuals store iron at higher levels than young people, and this contrast is more marked in elderly females than in male patients. This indicates that OP may also be influenced by age-related dysregulation of iron homeostasis. Sixthly, compared with purified compounds, crude plant extracts have greater compositional complexity and potential batch-to-batch variation, leading to lower controllability and reproducibility. Seventhly, LPON is a key metabolic feature of osteoporotic bone tissue. Levels of 4-hydroxynonenal, a key end product of LPON in bone tissue, are significantly elevated in patients with OP and gradually increase with age. Additionally, malondialdehyde levels in OP patients were also significantly higher than those in healthy individuals. Thus, LPO in bone tissue may serve as early biomarkers for OP. Finally, as key factors influencing bone metabolism, inflammation and immunogenicity have been implicated in ferroptosis across various diseases; however, no research has yet to elucidate whether these two processes regulate bone metabolism by ferroptosis.

To bridge these gaps, future investigations should focus on the following aspects: (1) Selective control of ferroptosis may be achieved by multiple approaches, including drug delivery, optimization of biodistribution and pharmacokinetics, and the selection of targets and mechanisms for regulating ferroptosis in specific contexts; (2) We need to determine the level of iron that leads to an irreversible threshold during ferroptosis, and whether iron kinetics ultimately dictate the outcome also requires clarification; (3) Comprehensive research is urgently required to elucidate the regulatory actions and functions of ferroptosis regulators across distinct cell types within bone tissue in order to provide broader prospects for therapeutic interventions in disease states; (4) Elucidating the crosstalk between ferroptosis and other PCD could offer critical perspectives on both the pathophysiological role of ferroptosis in OP and the development of therapeutic strategies; (5) There is a requirement for more efficient and cutting-edge technology development and precise identification to screen natural products, while simultaneously developing new drug delivery systems to enhance the bioavailability and specificity of natural products; (6) Future epidemiological research should determine whether a greater difference between iron and sex hormones may lead to greater bone loss; (7) Quality control methods such as chromatographic fingerprinting should be employed to enhance the controllability and reproducibility of the biological activity of crude plant extracts; In summary, this review gives a theoretical groundwork for exploring the mechanisms of ferroptosis and its interplay with OP, aiming to direct the development of natural product-based interventions for OP prevention and treatment.
